# Investigating the contribution of laboratory parameters on plasma neurofilament light chain levels in multiple sclerosis

**DOI:** 10.3389/fneur.2025.1620468

**Published:** 2025-09-17

**Authors:** Valerio Nicolella, Monica Gelzo, Carmela Polito, Giuseppina Affinito, Sveva Bagnasco, Raffaella Addesso, Gustavo Cernera, Rosa Sirica, Evelina La Civita, Mariano Fiorenza, Federica Novarella, Raffaele Palladino, Vincenzo Brescia Morra, Giuseppe Castaldo, Daniela Terracciano, Marcello Moccia

**Affiliations:** ^1^Department of Neuroscience, Reproductive Sciences and Odontostomatology, Federico II University of Naples, Naples, Italy; ^2^Department of Molecular Medicine and Medical Biotechnology, Federico II University of Naples, Naples, Italy; ^3^Centre for Advanced Biotechnology (CEINGE), Naples, Italy; ^4^Department of Translational Medical Sciences, Federico II University of Naples, Naples, Italy; ^5^Department of Public Health, Federico II University of Naples, Naples, Italy; ^6^Department of Precision Medicine in Medical, Surgical and Critical Care, University of Palermo, Palermo, Italy; ^7^Multiple Sclerosis Unit, Policlinico Federico II University Hospital, Naples, Italy

**Keywords:** multiple sclerosis, neurofilament, laboratory, metabolic status, biomarker

## Abstract

**Objective:**

To investigate the associations between several laboratory parameters and plasma neurofilament light chain (pNfL) in individuals with multiple sclerosis (MS), as well as their additional contribution to the established relationships between pNfL, demographics, and MS disability.

**Methods:**

In this cross-sectional study, we included 638 people with MS (PwMS) and evaluated pNfL (using fully automated chemiluminescent enzyme immunoassay), along with demographic, clinical and laboratory variables. Laboratory variables were preliminary selected using univariate linear regression models and multicollinearity analysis. A multivariate linear regression model was then employed to determine independent predictors of pNfL levels. Finally, we used linear regression models to explore the clinical utility of adjusting pNfL level.

**Results:**

On the multivariate linear regression model, higher pNfL was associated with older age (Coeff = 0.15; 95%CI = 0.04, 0.26; *p* = 0.007), presence of cardiovascular comorbidity (Coeff = 3.67; 95%CI = 0.82, 6.51; *p* = 0.012), higher alkaline phosphatase (ALP) (Coeff = 0.05; 95%CI = 0.01, 0.09; *p* = 0.19), higher lymphocytes’ fraction (Coeff = 0.20; 95%CI = 0.08,0.33; *p* = 0.001), lower blood proteins (Coeff = −4.02; 95%CI = -6.09, −1.96; *p* < 0.001), and lower hemoglobin (HB) (Coeff = −1.01; 95%CI = −1.73, −0.27; *p* = 0.007). We confirmed known association between higher pNfL and worse MS-related disability (Coeff = 2.23; 95%CI = 1.58, 2.87; <0.001), which did not significantly change after including selected laboratory variables (Coeff = 1.48; 95%CI = 0.72, 2.24; *p* < 0.001).

**Conclusion:**

Although laboratory markers of lymphocyte depletion and metabolic/nutritional status are correlated with pNfL levels, they do not modify its relationship with MS disability.

## Introduction

Neurofilaments are neuron-specific cytoskeletal proteins that are released after neuroaxonal damage in the cerebrospinal fluid (CSF) and, to a lesser extent, in the peripheral compartment1. The availability of newer immunoassays has allowed the measurement of neurofilament light chain (NfL) in different biological matrices, including blood (1). The possibility to measure plasma NfL (pNfL) holds potential in many neurological and psychiatric conditions (2, 3). NfL is elevated in central nervous system diseases and acute and chronic neuropathies, holding prognostic value (2, 4). In addition, NfL is associated with the severity of depression and with both subjective and objective assessments of substance use and substance use disorder severity, thus providing a biological framework for psychiatric diseases as well (5, 6).

Multiple sclerosis (MS) currently affects an estimated 1.89 million people worldwide, with a global prevalence of 23.9 cases per 100,000 population (7). In MS, pNfL has been gaining relevance to predict the risk of disease worsening (relapses, disability progression, and magnetic resonance imaging (MRI) lesions) and to monitor treatment response, which is a cornerstone to prevent disability (8–10).

However, the clinical application of pNfL is limited by the lack of specificity for MS-related mechanisms. For instance, pNfL significantly increases with age, according to physiological brain volume loss (11). More in general, any condition that affects brain health, such as cardiovascular risk factors and diseases, can lead to raised pNfL levels, independently from MS (12, 13). Also, pNfL levels can increase due to lower clearance (e.g., kidney dysfunction) or, by contrast, can decrease due to hemodilution (e.g., higher BMI) (14, 15). Consequently, various conditions can influence pNfL levels, raising questions about its reliability for clinical applications (16).

Many studies have reported alterations in biochemical parameters in people with MS (PwMS), prompting investigation into the potential utility of routinely-collected laboratory measures as disease biomarkers (17, 18). However, while routinely-collected laboratory measures do not hold specificity for neuro-axonal pathology, they could detect a wide range of pathological conditions affecting pNfL concentrations (19–21). When these conditions are accurately identified, they may provide valuable guidance for interpreting pNfL values (22, 23). In this context, our aim is to examine the associations between pNfL and these laboratory variables in PwMS and to assess their additional contribution beyond the known relationships between pNfL, demographic factors, and clinical features.

## Methods

### Study design and population

This is a secondary analysis of a previous cross-sectional study, conducted at the Federico II University Hospital (Naples, Italy), evaluating pNfL and its clinical correlates in PwMS. Hereby, we are including a large set of laboratory variables along with pNfL (21). We included consecutive people with a diagnosis of MS, from Sep to Nov 2023, regardless of age, disability status, or treatment status. Patients were asked to participate to the study at their scheduled neurological consultation and blood drawn. The full population is fully described elsewhere, and this study has been conducted on a subgroup with full availability of both pNfL and laboratory variables (24).

The study was approved by the Federico II Ethics Committee (332/21). All patients signed informed consent authorizing the use of anonymized data in line with data protection regulation (GDPR EU2016/679). The present study was performed in accordance with good clinical practice and Declaration of Helsinki.

### Demographics and clinical variables

Demographic and clinical variables were age, sex, height and weight [from which we calculated the body mass index (BMI)], smoking (ever or never smoker), cardiovascular comorbidities (high blood pressure, high cholesterol, diabetes, atrial fibrillation, stroke, coronary disease and/or related medications).

MS clinical variable was the expanded disability status scale (EDSS), a scale ranging from 0 (normal neurological disability) to 10 (death due to MS).

### NfL measurement

Fasting blood samples were obtained on the same day of the other clinical and laboratory assessments. Blood samples were centrifuged within 3 h after draw at 1100 rpm × 10 min, aliquoted into polypropylene tubes and stored at −80°C. pNfL levels were evaluated using fully automated chemiluminescent enzyme immunoassay (LUMIPULSE^®^, Fujirebio, Tokyo, Japan) and were expressed in picogram per milliliter (pg/mL).

### Laboratory variables

Fasting blood samples were obtained on the same day of the other clinical and laboratory assessments. Sera samples were obtained from blood samples in tubes with separation gels by centrifugation at 3500 rpm for 15 min. Serum parameters were determined by a Cobas prointegrated system (Cobas ISE, Cobas c503, Cobas e801, Roche Diagnostics). Hematological parameters were determined on blood sample in tubes with EDTA by ADVIA 2120i Hematology System (Siemens Healthcare GmbH).

### Statistical analysis

Study variables were described as mean and standard deviation or proportion, as appropriate. We performed univariate linear regression models to identify potential associations between pNfL and the full set of laboratory variables. Variables that reached a *p*-value less than 0.05 were later included in multivariable models. We also investigated the correlations between the selected variables for the presence of possible multicollinearity. In particular, we used Pearson’s correlation coefficients for normally distributed continuous variables and Spearman’s rank correlations for non-normally distributed continuous variables. If two variables were highly correlated (*r* ≥ 0.7), only one was retained in the final analysis, taking into consideration both biological plausibility and statistical relevance. Finally, a multiple linear regression model was employed to determine independent predictors of pNfL levels. To explore the clinical utility of adjusting pNfL level, we used linear regression models including pNfL as the dependent variable, EDSS as the independent variable, and, then, adjusted age and other laboratory variables identified as significant from the previous models as covariates.

We performed statistical analyses using Stata 18.0. Normal distribution of variables and residuals was checked with statistical and graphical methods. Results are reported as coefficients (Coeff), 95% confidence intervals (95%CI), and *p*-values, as appropriate, and were considered statistically significant for *p* < 0.05.

## Results

### Study population

We included 638 PwMS (age 49.73 ± 12.41 years; 65.55% females; pNfL 14.48 ± 14.81 pg./mL). Demographic, clinical, cognitive and laboratory variables are presented in Table 1.

**Table 1:** Demographic, clinical, laboratory variables and associations with pNfL.

Variable	*N* = 638	Univariate models			
		Coeff	95% CI		*p*-value
			Lower	Upper	
pNfL (pg/ml)	14.49 ± 14.81				
Age, years	49.73 ± 12.41	**0.29**	**0.20**	**0.37**	**<0.01**
Sex, females (%)	449 (65.55%)	2.09	0.24	4.43	0.079
Cardiovascular comorbidities (%)	199 (29.35%)	**6.58**	**4.16**	**8.99**	**<0.01**
Ever Smoking (%)	125 (18.25%)	−1.00	−3.99	1.87	0.494
BMI (*n* = 454)	25.01 ± 4.58	−0.10	−0.41	0.20	0.501
EDSS, median (range)	3.0 (1.0–8.0)	**2.23**	**1.58**	**2.87**	**<0.01**
Sodium (mmol/L)	140.71 ± 2.12	0.17	−0.38	0.73	0.542
Potassium (mmol/L)	4.19 ± 0.62	1.09	−0.83	3.00	0.266
Chlorum (mmol/L)	104.90 ± 4.91	0.07	−0.17	0.31	0.571
Calcium (mg/dl)	9.16 ± 0.53	0.63	−1.58	2.84	0.576
Phosphorus (mg/dl)	3.23 ± 0.51	0.60	1.68	2.89	0.603
Iron (μg/dL)	85.40 ± 33.28	**−0.05**	**−0.08**	**−0.01**	**0.010**
Ferritin (ng/ml)	105.60 ± 103.18	−0.00	−0.02	0.01	0.533
Glucose (mg/dl)	76.84 ± 16.78	0.05	−0.02	0.12	0.157
Urea (mg/dl)	36.18 ± 10.28	**0.13**	**0.02**	**0.25**	**0.020**
Creatinine (mg/dl)	0.98 ± 4.20	0.26	−0.20	0.54	0.064
Blood proteins (g/dl)	6.94 ± 0.56	**−4.85**	**−6.88**	**−2.82**	**<0.01**
Albumin (g/dl)	4.64 ± 0.48	−1.63	−4.12	0.87	0.201
Uric Acid (mg/dL)	4.65 ± 1.33	−0.21	−1.19	0.77	0.674
Total Bilirubin (mg/dL)	0.64 ± 0.47	2.42	−0.31	5.15	0.082
Direct Bilirubin (mg/dL)	0.26 ± 0.11	−0.02	−10.81	10.76	0.996
total cholesterol (mg/dL)	197.21 ± 40.73	−0.01	−0.04	0.02	0.366
LDL cholesterol (mg/dL)	120.71 ± 34.07	0.02	−0.02	0.06	0.287
HDL cholesterol (mg/dL)	55.33 ± 14.49	−0.02	0.10	0.06	0.641
Triglycerides (mg/dL)	108.45 ± 62.84	−0.01	−0.03	0.02	0.626
AST (U/L)	22.46 ± 9.47	0.09	−0.04	0.21	0.170
ALT (U/L)	24.28 ± 17.18	**−0.09**	**−0.17**	**−0.01**	**0.027**
GGT (U/L)	39.18 ± 48.48	−0.00	−0.03	0.02	0.725
ALP (U/L)	79.79 ± 28.87	**0.08**	**0.04**	**0.12**	**<0.01**
LDH (U/L)	209.74 ± 40.78	0.03	0.01	0.06	**0.019**
CK (U/L)	94.58 ± 63.70	−0.01	0.03	0.01	0.402
AMS (U/L)	66.12 ± 24.10	−0.02	0.07	0.03	0.409
CHE (U/L)	9137.25 ± 3860.06	**−0.00**	**−0.00**	**−0.00**	**0.019**
RBC (x10^6^/uL)	4.59 ± 0.60	1.13	−1.09	3.35	0.319
HB (g/dl)	17.53 ± 1.57	**−1.01**	**−1.71**	**−0.30**	**<0.01**
HCT (%)	40.92 ± 4.22	**−0.31**	**−0.57**	**−0.05**	**0.021**
MCV (fL)	87.71 ± 6.52	−0.15	−0.32	0.02	0.081
MCH (pg/cell)	29.53 ± 3.34	**0.44**	**0.11**	**0.77**	**0.010**
MCHC (g/dl)	33.48 ± 1.25	−0.87	−1.76	0.02	0.054
RDW (%)	13.84 ± 1.36	**1.04**	**0.22**	**1.86**	**0.013**
PLT (×10^3^/uL)	234.06 ± 66.19	0.01	−0.01	0.03	0.269
PCT (%)	0.24 ± 0.72	**16.06**	**0.37**	**31.75**	**0.045**
MPV (fL)	10.18 ± 1.39	0.67	−0.14	1.48	0.104
PDW (%)	29.10 ± 18.12	−0.04	0.10	0.02	0.216
WBC (×10^3^/uL)	20.31 ± 382.38	**0.01**	**0.01**	**0.01**	**<0.01**
Neutrophils’ fraction	68.95 ± 9.66	**−0.13**	**−0.24**	**−0.01**	**0.030**
Total neutrophils (×10^3^/uL)	19.46 ± 9.16	0.49	−0.25	1.24	0.193
Lymphocytes’ fraction	1.09 ± 0.62	**0.15**	**0.03**	**0.27**	**0.015**
Total lymphocytes (×10^3^/uL)	7.60 ± 2.35	**3.35**	**1.58**	**5.13**	**<0.01**
Monocytes’ fraction	0.41 ± 0.14	−0.22	−0.70	0.25	0.357
Total monocytes (×10^3^/uL)	2.66 ± 1.91	5.91	−1.90	13.72	0.138
Eosinophils’ fraction	0.15 ± 0.11	0.26	0.32	0.84	0.386
Total eosinophils (×10^3^/uL)	0.54 ± 0.30	**10.24**	**0.50**	**19.98**	**0.039**
Basophils’ fraction	0.03 ± 0.03	1.53	−2.20	5.27	0.420
Total basophils (×10^3^/uL)	3.90 ± 1.50	14.27	19.19	47.73	0.403

Table shows coefficients (Coeff), 95% confidence intervals (95% CI), and *p-*values from univariate linear regression models, including pNfL levels, as dependent variable, and each demographic, clinical and laboratory variable, in turn, as independent variable. Significant results (*p* < 0.05) are reported in bold. BMI, body mass index; EDSS, expanded disability status scale; LDL, low density lipoproteins, HDL, high density lipoproteins, AST, aspartate aminotransferase, ALT, alanine aminotransferase; GGT, Gamma-glutamyl transpeptidase; ALP, alkaline phosphatase; LDH, lactate dehydrogenase; CK, creatine kinase; AMS, amylase; CHE, cholinesterase; RBC, red blood cells, HB, hemoglobin; HCT, hematocrit; MCV, mean corpuscular volume; MCH, mean corpuscular hemoglobin, MCHC, mean corpuscular hemoglobin concentration; RDW, Red cell distribution width; PLT, platelets; PCT, plateletocrite; MPV, mean platelet volume; PDW, platelet distribution width; WBC, white blood cell.

### Univariate models for laboratory variables

On univariate linear regression models, higher pNfL was associated with older age (Coeff = 0.29; 95%CI = 0.20, 0.37; *p* < 0.01), presence of cardiovascular comorbidity (Coeff = 6.58; 95%CI = 4.16, 8.99; *p* < 0.01), higher urea (Coeff = 0.13; 95%CI = 0.02, 0.258.99; *p* = 0.020), higher alkaline phosphatase (ALP) levels (Coeff = 0.08; 95%CI = 0.04, 0.12; *p* < 0.01), higher lactate dehydrogenase (LDH) (Coeff = 0.03; 95%CI = 0.01, 0.06; *p* = 0.019), higher, mean corpuscular hemoglobin (MCH) (Coeff = 0.44; 95%CI = 0.11, 0.77; *p* = 0.010), higher red cell distribution width (RDW) (Coeff = 1.04; 95%CI = 0.22, 1.86; *p* = 0.013), higher plateletocrite (PCT) (Coeff = 16.06; 95%CI = 0.37,31.75; *p* = 0.045), higher white blood cell (WBC) (Coeff = 0.01; 95%CI = 0.01, 0.01; *p* < 0.01), higher lymphocytes’ fraction (Coeff = 0.15; 95%CI = 0.03, 0.27; *p* = 0.015), higher total lymphocytes (Coeff = 3.35;95%CI = 1.58, 5.13; *p* < 0.01), and higher total eosinophils (Coeff = 10.24; 95%CI = 0.50, 19.98; *p* = 0.039). Lower pNfL was associated with higher iron (Coeff = −0.05; 95%CI = −0.08, −0.01; *p =* 0.010), higher blood proteins (Coeff = −4.85; 95%CI = −6.88, −2.82; *p* < 0.01), higher alanine aminotransferase (ALT) levels (Coeff = −0.09; 95%CI = −0.17, −0.01; *p =* 0.027), higher cholinesterase (CHE) (Coeff = −0.00; 95%CI = −0.00, −0.04; *p =* 0.019), higher hemoglobin (HB) (Coeff = −1.01; 95%CI = −1.71, −0.30; *p =* 0.006), higher hematocrit (HCT) (Coeff = −0.31; 95%CI = −0.57, −0.05; *p =* 0.021), and higher neutrophils fraction (Coeff = −0.13; 95%CI = −0.24, −0.01; *p =* 0.030). Results of the univariate analyses are reported in Table 1.

### Multicollinearity analysis for laboratory variables

**Figure 1:**
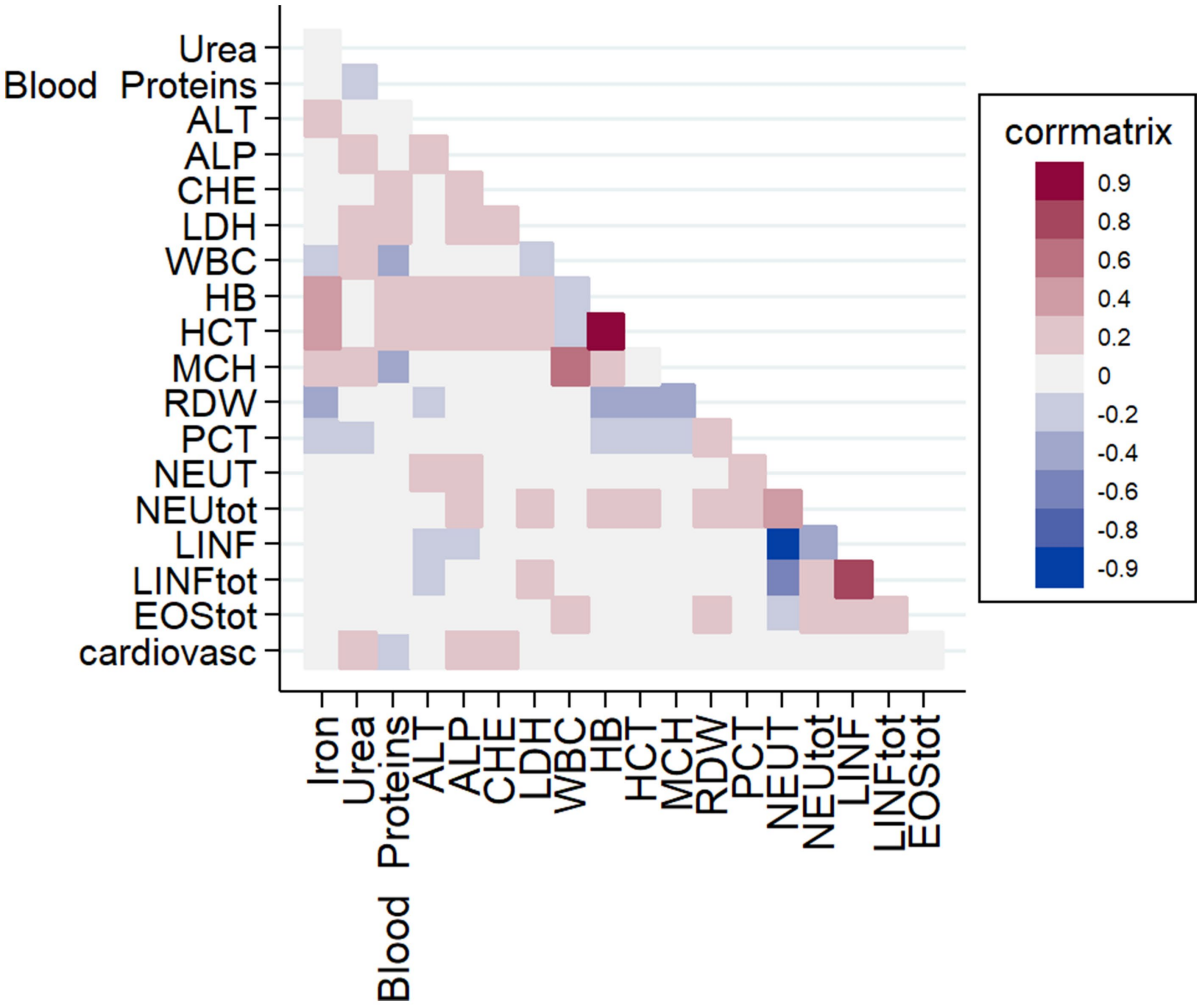
Heatmap of correlations between laboratory variables. Heatmap shows correlations between laboratory variables selected in univariate linear regression models. The color gradients provide the strength and direction of these associations. ALT, alanine aminotransferase; ALP, alkaline phosphatase; CHE, cholinesterase; LDH, lactate dehydrogenase; WBC, white blood cell; HB, hemoglobin; HCT, hematocrit; MCH, mean corpuscular hemoglobin; RDW, red cell distribution Width; PCT, plateletocrite; NEUT, neutrophils fraction; LINF, lymphocytes fraction; LINFtot, total neutrophils; EOStot, total eosinophils; cardiovasc, cardiovascular comorbidity.

Out of the variables selected from the univariate linear regression models (*p*-value less than 0.05), we found positive correlations between HB and HCT (*r* = 0.95) and between lymphocytes’ fraction and total lymphocytes (*r* = 0.80). Also, we found negative correlation between neutrophils’ fraction and lymphocytes’ fraction (*r* = −0.95) (Figure 1).

Based on the collinearity, on the results of univariate linear regression models (size of coefficients) and on the biological plausibility of associations, we preferred to retain HB over iron, HCT, MCH and RDW; lymphocytes’ fraction over WBC, neutrophils’ fraction, total lymphocytes and total eosinophils; blood proteins, ALP and LDH, over urea, ALT and CHE. Also, we excluded PCT due to wide confidence intervals.

### Multivariate model for laboratory variables

**Figure 2:**
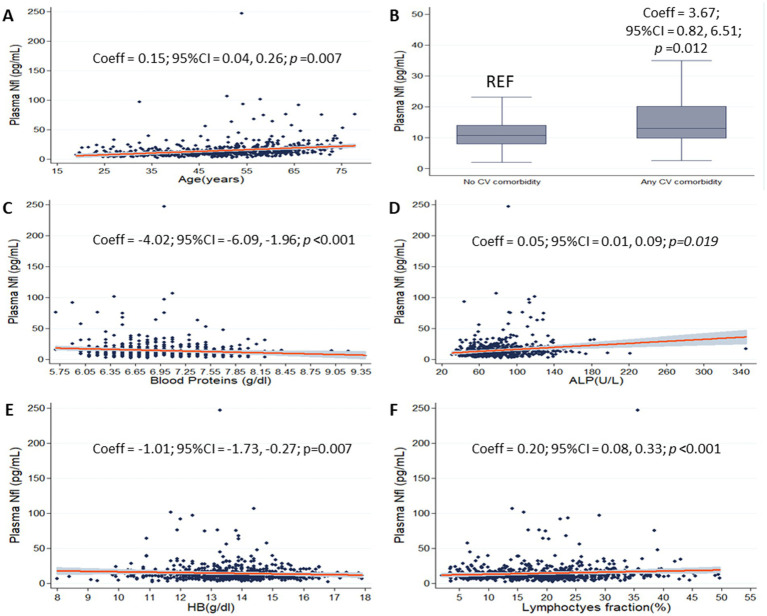
pNfL and selected laboratory variables. Scatter plots show the associations between plasma neurofilament light chain (pNfL) and age **(A)**, blood proteins **(C)**, alkaline phosphatase (ALP) **(D)**, hemoglobin (HB) **(E)** and lymphocytes’ fraction **(F)** (gray shades represent confidence intervals). Box plot shows the association between pNfL and cardiovascular comorbidity **(B)**. Coefficients (Coeff), 95% confidence intervals (95% CI), and *p* values are presented for significant associations.

On the multivariate linear regression model including the full set of variables as covariates (age, presence of cardiovascular comorbidity, blood proteins, ALP, LDH, HB and lymphocytes fraction, as selected by univariate models and subsequent multicollinearity analysis for laboratory variables), older age (Coeff = 0.15; 95%CI = 0.04, 0.26; *p* < 0.01), presence of cardiovascular comorbidity (Coeff = 3.67; 95%CI = 0.82, 6.51; *p* = 0.012), higher ALP (Coeff = 0.05; 95%CI = 0.01, 0.09; *p* = 0.19), and higher lymphocytes’ fraction (Coeff = 0.20; 95%CI = 0.08, 0.33; *p* = 0.001) were associated with higher pNfL. Also, lower pNfL was associated with higher blood proteins (Coeff = −4.02; 95%CI = −6.09, −1.96; *p* < 0.01), and higher HB (Coeff = −1.01; 95%CI = −1.73, −0.27; *p* < 0.01) were associated with higher pNfL (Figure 2). Table 2 shows the association between pNfl and its independent predictors selected from previous analyses.

**Table 2:** pNfL and selected laboratory variables.

Variable	Adjusted models			
	Coeff	95% CI		*p*-value
		Lower	Upper	
pNfL				
Age	**0.15**	**0.04**	**0.26**	**<0.01**
Cardiovascular comorbidity	**3.67**	**0.82**	**6.51**	**0.012**
Blood proteins	**−4.02**	**−6.09**	**−1.96**	**<0.01**
ALP	**0.05**	**0.01**	**0.09**	**0.019**
LDH	0.02	−0.00	0.05	0.101
HB	**−1.01**	**−1.73**	**−0.27**	**<0.01**
Lymphocytes’ fraction	**0.20**	**0.08**	**0.33**	**<0.01**

Table shows coefficients (Coeff), 95% confidence intervals (95% CI), and *p-*values from multiple linear regression models, including pNfL levels, as dependent variable, and age, sex, presence of cardiovascular comorbidity, blood proteins levels, ALP, LDH, HB and lymphocytes’ fraction as dependent variable. Significant results (*p* < 0.05) are reported in bold.

### Univariate and multivariate models for EDSS

On univariate linear regression model, higher pNfL was associated with higher EDSS (Coeff = 2.23; 95%CI = 1.58, 2.87; <0.01). On multivariate linear regression model including age as covariate, we confirmed the association between higher pNfL levels and higher EDSS (Coeff = 1.56; 95%CI = 0.83, 2.89; <0.01). On multivariate linear regression model including the full set of variables as covariates (age, presence of cardiovascular comorbidity, blood proteins, ALP, LDH, HB and lymphocytes fraction, as selected by univariate models and subsequent multicollinearity analysis for laboratory variables), higher pNfL levels remained associated with higher EDSS, in the absence of significant changes in the correlation coefficient (Coeff = 1.48; 95%CI = 0.72, 2.24; *p* < 0.01). Also, we confirmed the associations between higher pNfL and presence of cardiovascular comorbidity (Coeff = 3.77; 95%CI = 0.96, 6.58; *p* = 0.009), higher lymphocytes’ fraction (Coeff = 0.21; 95% = 0.09, 0.34, *p* = 0.001) and between lower pNfL and higher blood proteins (Coeff = −3.76; 95%CI = −5.81, −1.72; *p* < 0.01) and higher HB (Coeff = −1.02; 95%CI = −1.74, −0.30; *p* < 0.01).

## Discussion

Our study showed that several laboratory parameters were significantly and independently associated with pNfL levels in MS, likely reflecting both overall metabolic and nutritional status (e.g., blood proteins, ALP, LDH, and hemoglobin) and the MS-specific response to immunosuppressive therapies (e.g., lymphocyte counts). While these associations might prove helpful in identifying pathological states affecting pNfL levels, its clinical utility remained unaffected.

Looking at laboratory markers of metabolic function, we found that higher pNfL levels were associated with higher ALP levels and lower blood proteins. Both ALP and blood proteins reflect liver function and, more in general, the nutritional status of individuals (25–27). Ladang et al. (28) and Pratt et al. (29) found that higher blood NfL levels were associated with more severe stages of muscular loss and frailty. In keep with this, higher pNfL levels could reflect more disabling disease, and, in turn, worse nutritional status (30), with reduced blood proteins and loss of muscle structure, with subsequently increased ALP (31, 32). Similar consideration could apply to HB, reflecting the overall iron metabolism and related functional status (as also shown by associations in univariate models with iron, HCT, MCH and RDW) (33–37).

Several studies investigated the relationship between nutritional status and pNfL (19–21). Nilsson et al. (19) reported that in patients with anorexia nervosa (a condition characterized by severe nutritional alterations), pNfL levels were significantly increased. This suggests that a compromised nutritional status may be associated with neuronal damage detectable through this biomarker. Thota et al. (20) highlighted that metabolic alterations related to nutritional status, such as impaired glycaemic control and insulin resistance, were associated with variations in pNfL levels in middle-aged adults, suggesting a link between metabolism and neurodegeneration. Wang et al. (21) found that an higher intake of polyunsaturated fatty acids (PUFAs) is associated with lower sNfL levels, which may reflect a reduced extent of neuroaxonal injury. Altogether, these studies demonstrated how various aspects of nutritional status can influence plasma NfL levels, highlighting the need for an integrated evaluation also of the laboratory parameters in the context of potential clinical applications.

Inflammation is a major driver of the MS pathophysiology and, thus, most MS treatments are immunosuppressants and reduce lymphocyte levels (37). In our previous study on the same population, we showed lower levels of pNfL in PwMS treated with DMTs when compared with no treatment, and in PwMS treated with high-efficacy DMTs when compared with low-moderate efficacy DMTs (24). This is in line with the current body of literature, showing that the reduction of pNfL mirrors the level of treatment efficacy (8, 37). Hereby, we found that higher levels of pNfL were associated with higher lymphocytes’ fraction, possibly reflecting the use of medications not affecting lymphocyte levels (i.e., low-efficacy DMTs) (38, 39).

Additionally, we confirmed that higher pNfL levels are associated with both older age and the presence of cardiovascular comorbidities (12, 13). Interestingly, in our previous analysis of the same cohort, the associations between pNfL and older age as well as between pNfL and cardiovascular comorbidities appeared interdependent, resulting into mutually exclusive effects when modeled together. In the current analysis, however, after accounting for laboratory variables, both age and cardiovascular comorbidity remained independently associated with pNfL, suggesting that the interplay between age and comorbidities is more complex than previously understood (40, 41).

Regarding clinical features, we have already analyzed the associations between pNfL and the clinical characteristics of MS in the full study population (24). Here, we confirmed a significant relationship between higher pNfL levels and greater disability (EDSS) (8–10). Notably, when age was added as a covariate, the association coefficient decreased from 2.23 to 1.56, suggesting that age-related disease progression may partly account for this relationship. Furthermore, including all laboratory variables in the final multiple regression model resulted in a minimal further change (coefficient = 1.48), indicating that these selected laboratory variables do not substantially influence pNfL levels or their clinical associations in this neurological disease population compared to age and cardiovascular comorbidities (42–44).

A limitation of this study is the inclusion of a population of PwMS only; therefore, our findings need to be replicated in control groups and in other neurological and psychiatric diseases (2, 3). Also, we evaluated clinical utility by using the EDSS, which was the strongest clinical correlate of pNfL in our previous study. Of course, the observed pNfL concentrations might have been affected by unmeasured factors not accounted for in the present study (2, 3, 22). Furthermore, we did not conduct analyses on the association between clinical and laboratory variables, as these have already been extensively explored in previous studies (18, 45). Additionally, since our final models were derived solely using linear and correlation analyses, we may have inadvertently excluded variables that exhibit non-linear relationships with pNfL levels (12).

In conclusion, our study demonstrated that, in PwMS, pNfL levels not only serve as biomarkers of disability, but are also independently affected by various laboratory markers, including lymphocyte depletion and metabolic and nutritional status. The interpretation of NfL should carefully take into account not only the clinical suspect, but also the framework of general laboratory analyses.

## Data Availability

The raw data supporting the conclusions of this article will be made available by the authors, without undue reservation.

## References

[1] Gaetani L Blennow K Calabresi P Di Filippo M Parnetti L Zetterberg H. Neurofilament light chain as a biomarker in neurological disorders. J Neurol Neurosurg Psychiatry. (2019) 90:870–81. doi: 10.1136/jnnp-2018-320106, PMID: 30967444

[2] Khalil M Teunissen CE Lehmann S Otto M Piehl F Ziemssen T. Neurofilaments as biomarkers in neurological disorders – towards clinical application. Nat Rev Neurol. (2024) 20:269–87. doi: 10.1038/s41582-024-00955-x, PMID: 38609644

[3] Bavato F Barro C Schnider LK Simrén J Zetterberg H Seifritz E. Introducing neurofilament light chain measure in psychiatry: current evidence, opportunities, and pitfalls. Mol Psychiatry. (2024) 29:2543–59. doi: 10.1038/s41380-024-02524-6, PMID: 38503931 PMC11412913

[4] Romano A Primiano G Antonini G Ceccanti M Fenu S Forcina F. Serum neurofilament light chain: a promising early diagnostic biomarker for hereditary transthyretin amyloidosis? Eur J Neurol. (2024) 31:e16070. doi: 10.1111/ene.16070, PMID: 37724995 PMC11235699

[5] Zhang K Cheng M Yang P Hu Y Liang X Li M. Association between serum neurofilament light chains and depression: a cross-sectional study based on NHANES 2013-2014 database. J Affect Disord. (2025) 368:591–8. doi: 10.1016/j.jad.2024.09.063, PMID: 39277033

[6] Li Y Duan R Gong Z Jing L Zhang T Zhang Y. Neurofilament light chain is a promising biomarker in alcohol dependence. Front Psych. (2021) 12:754969. doi: 10.3389/fpsyt.2021.754969, PMID: 34867542 PMC8637455

[7] Khan G Hashim MJ. Epidemiology of multiple sclerosis: global, regional, national and sub-National-Level Estimates and future projections. J Epidemiol Glob Health. (2025) 15:21. doi: 10.1007/s44197-025-00353-6, PMID: 39928193 PMC11811362

[8] Kuhle J Kropshofer H Haering DA Kundu U Meinert R Barro C. Blood neurofilament light chain as a biomarker of MS disease activity and treatment response. Neurology. (2019) 92:E1007–15. doi: 10.1212/WNL.0000000000007032, PMID: 30737333 PMC6442011

[9] Abdelhak A Benkert P Schaedelin S Boscardin WJ Cordano C Oechtering J. Neurofilament light chain elevation and disability progression in multiple sclerosis. JAMA Neurol. (2023) 80:1317–25. doi: 10.1001/jamaneurol.2023.3997, PMID: 37930670 PMC10628837

[10] Nicolella V Varelli M Fasano S Sirica R Polito C Saviano A. Clinical application of age-derived cut-offs for plasma neurofilament light chain in multiple sclerosis. J Neurol. (2025) 272:495. doi: 10.1007/s00415-025-13223-9, PMID: 40632270

[11] Khalil M Pirpamer L Hofer E Voortman MM Barro C Leppert D. Serum neurofilament light levels in normal aging and their association with morphologic brain changes. Nat Commun. (2020) 11:812. doi: 10.1038/s41467-020-14612-6, PMID: 32041951 PMC7010701

[12] Polymeris AA Coslovksy M Aeschbacher S Sinnecker T Benkert P Kobza R. Serum neurofilament light in atrial fibrillation: clinical, neuroimaging and cognitive correlates. Brain Commun. (2020) 2:166. doi: 10.1093/braincomms/fcaa166, PMID: 33381755 PMC7753055

[13] Korley FK Goldstick J Mastali M Van Eyk JE Barsan W Meurer WJ. Serum NfL (Neurofilament light chain) levels and incident stroke in adults with diabetes mellitus. Stroke. (2019) 50:1669–75. doi: 10.1161/STROKEAHA.119.024941, PMID: 31138085 PMC6591022

[14] Dittrich A Ashton NJ Zetterberg H Blennow K Zettergren A Simrén J. Association of Chronic Kidney Disease with Plasma NfL and other biomarkers of neurodegeneration. Neurology. (2023) 101:e277–88. doi: 10.1212/WNL.0000000000207419, PMID: 37225431 PMC10382262

[15] Tortosa-Carreres J Cubas-Núñez L Sanz MT Castillo-Villalba J Gasqué-Rubio R Carratalá-Boscá S. Renal function’s impact on serum neurofilament levels in patients with multiple sclerosis: an exploratory analysis. Neurol Sci. (2024) 46:845–53. doi: 10.1007/s10072-024-07772-6 39307881

[16] Hu YH Meirelles O Shiroma EJ Satizabal CL Seshadri S Tracy RP. Peripheral risk factors and their role in biomarker-based screening for dementia in the community. Alzheimers Dement. (2024) 20:8556–65. doi: 10.1002/alz.14293, PMID: 39718159 PMC11667489

[17] Porter L Shoushtarizadeh A Jelinek GA Brown CR Lim CK de Livera AM. Metabolomic biomarkers of multiple sclerosis: a systematic review. Front Mol Biosci. (2020) 7:574133. doi: 10.3389/fmolb.2020.574133, PMID: 33381517 PMC7768024

[18] Moccia M Lanzillo R Costabile T Russo C Carotenuto A Sasso G. Uric acid in relapsing-remitting multiple sclerosis: a 2-year longitudinal study. J Neurol. (2015) 262:961–7. doi: 10.1007/s00415-015-7666-y, PMID: 25673130

[19] Nilsson IAK Millischer V Karrenbauer VD Juréus A Salehi AM Norring C. Plasma neurofilament light chain concentration is increased in anorexia nervosa. Transl Psychiatry. (2019) 9:180. doi: 10.1038/s41398-019-0518-2, PMID: 31371701 PMC6675786

[20] Thota RN Chatterjee P Pedrini S Hone E Ferguson JJA Garg ML. Association of Plasma Neurofilament Light Chain with Glycaemic Control and Insulin Resistance in middle-aged adults. Front Endocrinol. (2022) 13:13. doi: 10.3389/fendo.2022.915449, PMID: 35795150 PMC9251066

[21] Wang Y Wang J Wang L. Association between polyunsaturated fatty acids intake and serum neurofilament light chain concentrations in American adults: a cross-sectional study. Front Nutr. (2025) 12:1608211. doi: 10.3389/fnut.2025.1608211, PMID: 40717996 PMC12289513

[22] Ramanathan M. Non-neurological factors associated with serum neurofilament levels in the United States population. Ann Clin Transl Neurol. (2024) 11:1347–58. doi: 10.1002/acn3.52054, PMID: 38586941 PMC11093254

[23] Chen X Lin Y Wei K. Elevated serum Neurofilament light chain levels are associated with all-cause mortality: evidence from National Health and nutrition examination survey. J Gerontol Series A. (2023) 78:2382–6. doi: 10.1093/gerona/glad058, PMID: 36811342

[24] Nicolella V Fiorenza M Monteiro I Novarella F Sirica R D'Angelo M. Clinical utility of the Lumipulse™ immunoassay for plasma neurofilament light chain in multiple sclerosis. J Neurol Sci. (2024) 463:123115. doi: 10.1016/j.jns.2024.123115, PMID: 38964268

[25] Makris K Mousa C Cavalier E. Alkaline phosphatases: biochemistry, functions, and measurement. Calcif Tissue Int. (2022) 112:233–42. doi: 10.1007/s00223-022-01048-x, PMID: 36571614

[26] Allen SL Quinlan JI Dhaliwal A Armstrong MJ Elsharkawy AM Greig CA. Sarcopenia in chronic liver disease: mechanisms and countermeasures. Am J Physiol. (2021) 320:G241–57. doi: 10.1152/ajpgi.00373.2020, PMID: 33236953 PMC8609568

[27] Osserman EF Takatsuki K. The plasma proteins in liver disease. Med Clin North Am. (1963) 47:679–710. doi: 10.1016/S0025-7125(16)33572-6, PMID: 13940565

[28] Ladang A Kovacs S Lengelé L Locquet M Beaudart C Reginster JY. Neurofilament-light chains (NF-L), a biomarker of neuronal damage, is increased in patients with severe sarcopenia: results of the SarcoPhAge study. Aging Clin Exp Res. (2023) 35:2029–37. doi: 10.1007/s40520-023-02521-9, PMID: 37581861 PMC10520189

[29] Pratt J De Vito G Segurado R Pessanha L Dolan J Narici M. Plasma neurofilament light levels associate with muscle mass and strength in middle-aged and older adults: findings from GenoFit. J Cachexia Sarcopenia Muscle. (2022) 13:1811–20. doi: 10.1002/jcsm.12979, PMID: 35415973 PMC9178157

[30] Bock M Steffen F Zipp F Bittner S. Impact of dietary intervention on serum Neurofilament light chain in multiple sclerosis. Neurol Neuroimmunol Neuroinflamm. (2022) 9:1102. doi: 10.1212/NXI.0000000000001102, PMID: 34764215 PMC8587737

[31] Yuksel H Balaban M Tan OO Mungan S. Sarcopenia in patients with multiple sclerosis. Mult Scler Relat Disord. (2022) 58:103471. doi: 10.1016/j.msard.2021.103471, PMID: 34998245

[32] Gaemelke T Pedersen IS Dalgas U Hvid LG. Sarcopenia in older people with multiple sclerosis: a cross-sectional study. Mult Scler Relat Disord. (2025) 93:106190. doi: 10.1016/j.msard.2024.106190, PMID: 39631136

[33] Koudriavtseva T Renna R Plantone D Mandoj C Piattella MC Giannarelli D. Association between Anemia and multiple sclerosis. Eur Neurol. (2015) 73:233–7. doi: 10.1159/000381212, PMID: 25823947

[34] Peters N. Neurofilament light chain as a biomarker in cerebral small-vessel disease. Mol Diagn Ther. (2022) 26:1–6. doi: 10.1007/s40291-021-00566-y, PMID: 34825310

[35] Zhang Z Gao S Dong M Luo J Xu C Wen W. Relationship between red blood cell indices (MCV, MCH, and MCHC) and major adverse cardiovascular events in anemic and nonanemic patients with acute coronary syndrome. Dis Markers. (2022) 2022:1–12. doi: 10.1155/2022/2193343, PMID: 36393972 PMC9649320

[36] Heo J Youk TM Seo KD. Anemia is a risk factor for the development of ischemic stroke and post-stroke mortality. J Clin Med. (2021) 10:2556. doi: 10.3390/jcm10122556, PMID: 34207841 PMC8226740

[37] Hemmer B Kerschensteiner M Korn T. Role of the innate and adaptive immune responses in the course of multiple sclerosis. Lancet Neurol. (2015) 14:406–19. doi: 10.1016/S1474-4422(14)70305-9, PMID: 25792099

[38] Benkert P Meier S Schaedelin S Manouchehrinia A Yaldizli Ö Maceski A. Serum neurofilament light chain for individual prognostication of disease activity in people with multiple sclerosis: a retrospective modelling and validation study. Lancet Neurol. (2022) 21:246–57. doi: 10.1016/S1474-4422(22)00009-6, PMID: 35182510

[39] Gelibter S Pisa M Croese T Dalla Costa G Orrico M Preziosa P. Neutrophil-to-lymphocyte ratio: a marker of neuro-inflammation in multiple sclerosis? J Neurol. (2021) 268:717–23. doi: 10.1007/s00415-020-10322-7, PMID: 33389030

[40] Simrén J Andreasson U Gobom J Suarez Calvet M Borroni B Gillberg C. Establishment of reference values for plasma neurofilament light based on healthy individuals aged 5-90 years. Brain Commun. (2022) 4:174. doi: 10.1093/braincomms/fcac174, PMID: 35865350 PMC9297091

[41] Petruzzo M Reia A Maniscalco GT Luiso F Lanzillo R Russo CV. The Framingham cardiovascular risk score and 5-year progression of multiple sclerosis. Eur J Neurol. (2021) 28:893–900. doi: 10.1111/ene.14608, PMID: 33091222

[42] Trojano M Liguori M Bosco Zimatore G Bugarini R Avolio C Paolicelli D. Age-related disability in multiple sclerosis. Ann Neurol. (2002) 51:475–80. doi: 10.1002/ana.10147, PMID: 11921053

[43] Manouchehrinia A Westerlind H Kingwell E Zhu F Carruthers R Ramanujam R. Age related multiple sclerosis severity score: disability ranked by age. Mult Scler J. (2017) 23:1938–46. doi: 10.1177/1352458517690618, PMID: 28155580 PMC5700773

[44] Husseini L Jung J Boess N Kruse N Nessler S Stadelmann C. Neurofilament light chain serum levels mirror age and disability in secondary progressive multiple sclerosis. Neurol Neuroimmunol Neuroinflamm. (2024) 11:279. doi: 10.1212/NXI.0000000000200279 PMC11256980 38991171

[45] Spiezia AL Falco F Manganelli A Carotenuto A Petracca M Novarella F. Low serum 25-hydroxy-vitamin D levels are associated with cognitive impairment in multiple sclerosis. Mult Scler Relat Disord. (2023) 79:105044. doi: 10.1016/j.msard.2023.105044, PMID: 37837668

